# Nanometer-Precision Tracking of Adipocyte Dynamics via Single Lipid Droplet Whispering-Gallery Optical Resonances

**DOI:** 10.1021/acssensors.5c03272

**Published:** 2025-12-29

**Authors:** Rok Podlipec, Ana Krišelj, Maja Zorc, Petra Matjan Štefin, Siegfried Usaar, Matjaž Humar

**Affiliations:** † Department of Condensed Matter Physics, 61790 Jozef Stefan Institute, Jamova 39, SI-1000 Ljubljana, Slovenia; ‡ Department of Biochemistry and Molecular and Structural Biology, Jozef Stefan Institute, Jamova cesta 39, SI-1000 Ljubljana, Slovenia; § Jozef Stefan International Postgraduate School, Jamova cesta 39, Sl-1000 Ljubljana, Slovenia; ∥ Research Unit Adipocytes & Metabolism (ADM), Helmholtz Diabetes Center, Helmholtz Zentrum München, 85764 Neuherberg, Germany; ⊥ Research Center for Environmental Health GmbH, 85764 Neuherberg, Germany; # German Center for Diabetes Research (DZD), 85764 Neuherberg, Germany; ∇ Faculty of Mathematics and Physics, University of Ljubljana, Jadranska 19, SI-1000 Ljubljana, Slovenia; ○ CENN Nanocenter, Jamova 39, SI-1000 Ljubljana, Slovenia

**Keywords:** adipocyte dynamics, lipid droplets, whispering gallery modes, optical resonances, lipolysis

## Abstract

Biophotonics—and more recently, biointegrated photonics—offer transformative tools for probing cellular processes with unprecedented precision. Among these, whispering-gallery-mode (WGM) resonators (optical microcavities formed in spherical structures) have emerged as powerful biosensors and intracellular barcodes. Lipid droplets (LDs), with their high refractive index and intrinsic spherical geometry, are ideal candidates for supporting intracellular lasing. Although lasing in LDs has been previously demonstrated, it has not yet been harnessed to study live-cell biology. Here, we report the first use of WGM resonances in LDs of live primary adipocytes, employing a continuous-wave (CW) laser at powers below the biological damage threshold. By measuring these resonances, we achieved nanometer-scale precision in size estimation, enabling real-time observation of rapid LD dynamics and deformations on the minute scale—far beyond the spatiotemporal resolution of conventional microscopy. We systematically characterized this photonic sensing approach, demonstrating its ability to resolve adipocyte heterogeneity, monitor lipolytic responses to forskolin and isoproterenol, and detect early signs of cell viability loss—well before conventional assays. This proof-of-concept establishes intracellular LD WGM resonances as a robust platform for investigating live single-cell metabolism. The technique enables rapid, cost-effective assessment of adipocyte function, reveals cell-to-cell variability obscured by bulk assays, and lays the foundation for high-throughput analysis of metabolism- and obesity-related diseases at both the cellular and tissue levels.

## Graphical abstract

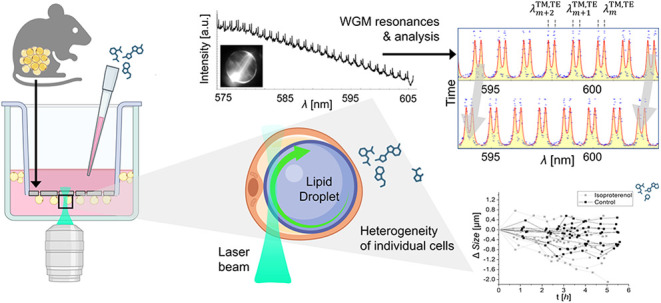

Biointegrated photonics and biophotonics are rapidly emerging fields in cellular sensing, leveraging advanced optical technologies to study and manipulate biological processes with exceptional precision.^1^ One of the highly promising biosensing strategy employs whispering-gallery-mode (WGM) resonators,^2,3^ which exploit optical resonances in spherical objects, such as microspheres and microdroplets. Light waves induced and propagated within these cavities undergo continuous internal reflection along the concave surface, producing constructive interference.^4^ The resonant conditions—capable of achieving extremely high quality (Q) factors—depend on the refractive index contrast between the cavity and its surrounding environment and exponentially on the microcavity size.^5^ These unique properties have enabled a broad spectrum of applications in biological and physical sensing,^4,6–8^ offering remarkable sensitivity for detecting subtle changes at the single-cell^9^ and single-molecule levels.^10–12^ Applications range from detecting single proteins and silica nanobead binding,^10^ to plasmon-enhanced sensing with nanorods for short nucleic acid strands,^11^ and even single-virus tracking.^13^ More recently, significant attention has been directed toward intracellular probing techniques for cell tagging, barcoding, and tracking,^14–19^ cavity-enhanced bioluminescence,^20^ and investigations into cellular (patho)­physiology, including cardiac contractility^21^ and molecular binding.^22^

Despite the promise of this emerging research frontier, intracellular studies utilizing biointegrated microlasers remain limited, particularly in addressing biologically relevant questions such as complex cell heterogeneity in disease contexts.^9^ One highly underexplored yet potentially transformative approach involves lasing of endogenous cellular structures. Lipid droplets (LDs), owing to their high sphericity and elevated refractive index relative to the surrounding cytoplasm, are capable of supporting intracellular lasing.^14^ Lasing in LDs is feasible for droplets larger than approximately 25 μm where radiative losses are sufficiently minimized. This could make adipose tissue (AT)—which contains LDs of the sizes ranging up to 100 μm and more in mature adipocytes^23^—an ideal candidate for such applications. However, to date, intracellular lasing in LDs has not been applied to biological studies of adipocytes.

LDs primarily serve as reservoirs for lipids—essential components for maintaining metabolic energy reserves and supplying lipids for cellular membranes.^24^ Although central to adipocyte function, the role of LD metabolism and its dysregulation in metabolic diseases remains underexplored.^25^ It is now recognized that LDs play multiple roles in systemic homeostasis and obesity-related pathologies,^26–29^ extending far beyond fat storage. They act as dynamic hubs for lipid management, integrating metabolic signals and lipid fluxes with diverse cellular homeostatic and stress responses.^30^ Despite their critical role in AT regulation, the dynamic nature and biological activity of LDs are still poorly understood.^31^ Current data are largely limited to single-cell gene expression analyses^32^ and bulk functional assays, such as lipolysis kits applied to large populations of adipocytes.^33^ These approaches fail to capture the heterogeneity of the LD function and dynamics both within and between individual cells. Moreover, averaging LD behavior across cell populations obscures the contributions of individual organelles, limiting our understanding of their nuanced/complex roles.

To overcome these limitations, novel optical methods are needed to assess LD dynamics in living, single primary adipocytes from animal or human sources. In recent years, several experimental approaches have emerged, including morphology-oriented live-cell and single-cell imaging of LDs using fluorescence microscopy,^34,35^ the development of advanced fluorescent probes,^36^ label-free Raman microscopy,^37,38^ and machine learning–based tools and analysis.^39^ Although these techniques have enabled the study of metabolic processes at cellular and subcellular levels previously inaccessible,^40^ they remain constrained by the optical resolution limit of ∼ 250 nm (Rayleigh criterion), which prevents the detection of morphological changes below this threshold. Limited spatial resolution also restricts temporal resolution, making it difficult to capture rapid, nanometer-scale changes in LD size—processes critical for understanding early metabolic shifts and predicting long-term outcomes of LD activity. LD growth (e.g., induced lipogenesis^41^) and shrinkage (e.g., induced lipolysis^42^) are often slow, occurring over hours, days, or even weeks before substantial size changes become detectable via live-cell microscopy.^43^ Achieving nanometer precision in LD size measurements would enable the near-instantaneous detection of growth or shrinkage, offering valuable insights into short-term metabolic dynamics.

To address these challenges, we present and characterize, for the first time, WGM optical resonances in natural intracellular lipid droplets as a means of biological sensing in adipocytes. Building on our previously established WGM-based methodology for embedded lasing in live cells,^14^ this approach offers exceptionally high-spatial (nanometer) and temporal (minute-scale) resolution for precise tracking and investigating individual LD size and dynamics. While our previous study focused solely on demonstrating lasing in adipocytes, here we systematically evaluate the methodological, physical, and biological aspects of photonic sensing in live adipocytes, highlighting both its strengths and limitations. This includes a detailed assessment of potential laser-induced effects and the optimization of laser parameters, which identify low-power continuous-wave (CW) lasers as the most suitable sources for biological applications. Rather than focusing on detailed biological interpretations, our primary aim is to establish a robust methodological foundation for future studies. These findings pave the way for advanced metabolic investigations into the highly heterogeneous and dynamic nature of adipocytes and adipose tissue.

## Materials and Methods

### Materials

Visceral fat (isolated from FVB/N female mice), 1× phosphate buffer saline (PBS, Gibco), Dulbecco’s Modified Eagle’s medium (DMEM, Gibco), Collagenase IV (Gibco), fatty acid-free bovine serum albumin (BSA FAF, Sigma-Aldrich), fetal bovine serum (FBS, Gibco), Pyrromethene 597 (Exciton Luxottica), verapamil (Spirochrome), SYTOX Deep Red (Thermo Fischer Scientific), CellMask Deep Red (Thermo Fischer Scientific), forskolin (Sigma-Aldrich), and triacsin (Cayman).

### Animals

Mice were used in accordance with the Administration of the Republic of Slovenia for food safety, veterinary, and plant protection (permit number: U34401-5/2022/15). Procedures for animal care and experiments were in accordance with the “Guide for Care and Use in Laboratory Animals.”

### Methods

#### Adipocyte Isolation

Visceral AT was isolated from FVB/N female mice. After the removal of larger veins, AT was cut into smaller pieces (1–2 mm^3^) and washed three times in cold PBS. Pieces were transferred in a 25 mL centrifuge tube with DMEM, complemented with penicillin (100 IU/mL), streptomycin (100 μg/mL), 1 mg/mL Collagenase Type IV, and 1% (w/v) BSA (FAF). A 2 mL portion of the collagenase solution was used per 1 g of fat tissue. Minced fat tissue was then incubated at 37 °C for 30–40 min and gently shaken every 10 min to allow for adequate disaggregation and isolation of individual adipocytes, preserving their viability. After digestion was complete, the suspension was mixed on a vortex mixer for 10 s to release the remaining cells from the tissue and then passed through a sieve with 100 μm pores. Collagenase was neutralized with the same volume of FBS. The cell suspension was then allowed to separate into layers at 37 °C for 15 min due to density differences. The top lipid layer and the bottom medium layer were discarded, followed by carefully pipetting of mature adipocytes into a fresh centrifuge tube, where they were washed 3 times with cell culture medium (DMEM, complemented with penicillin (100 IU/mL), streptomycin (100 μg/mL), 4 mM l-Glutamine, 10% FBS, 0.5 μg/mL insulin, and 0.4 ng/mL dexamethasone). Mature adipocytes were cultured in 12-well plates at 37 °C and 5% CO_2_. Each well in a 12-well plate was filled with 1 mL of cell culture medium and 200 μL of a dense suspension of isolated adipocytes. Medium without insulin and dexamethasone was used for further experiments.

#### Adipocyte Labeling for Optical Sensing and Dynamics Studies

The cell suspension was mixed with 1.5 μg/mL (4 μm) pyrromethene 597 BODIPY laser dye for labeling of LDs to enable lasing applications. After a few hours of incubation at 37 °C and 5% CO_2_, the cell media containing LD labels were exchanged with the cell media containing CellMask Deep Red (2.5 μg/mL, 2000× diluted stock solution) and SYTOX Red (5 nM, 1000× diluted stock solution) for 15 min incubated at 37 °C and 5% CO_2_. The volume of 1 mL suspension was then exchanged four times with insulin-free cell media to remove nonlabeled stains. For the optimal experimental conditions, ceiling translucent transwell inserts (3 μm pores, Falcon) were carefully immersed and fixed inside the 12-well plates, where a significant part of the densely populated floating adipocytes remained confined beneath the porous surface (Figure 1). For studying adipocyte dynamics and lipolytic metabolic activity in response to external stimuli, a cell medium containing lipolytic agents, forskolin, and isoproterenol was gently poured and mixed in the transwell chamber over time. A combination of 20 μm forskolin and 5 μm triacsin was used to stimulate lipolysis^44^ and prevent the regeneration of triglycerides.^45^ In contrast, 10 μm isoproterenol, acting through a different mechanism of cellular signaling,^46^ was used to stimulate lipolysis. Additionally, 5 μm verapamil was added to forskolin solution to check possible lipolytic change by blocking calcium (influx) channels^47^ important for regulation of the lipid metabolism.^48,49^ For control samples, no lipolytic agents were added to the transwell chamber. A 12-well plate with transwell inserts was then transferred into the stage-top incubator (H301-K-FRAME, Okolab) mounted on an inverted microscope (Nikon ECLIPSE Ti2) for LD dynamics studies of live adipocytes. The experiment was run for a total of 6 to 24 h, with lipolytic agents being added 2–3 h after the start.

**Figure 1:**
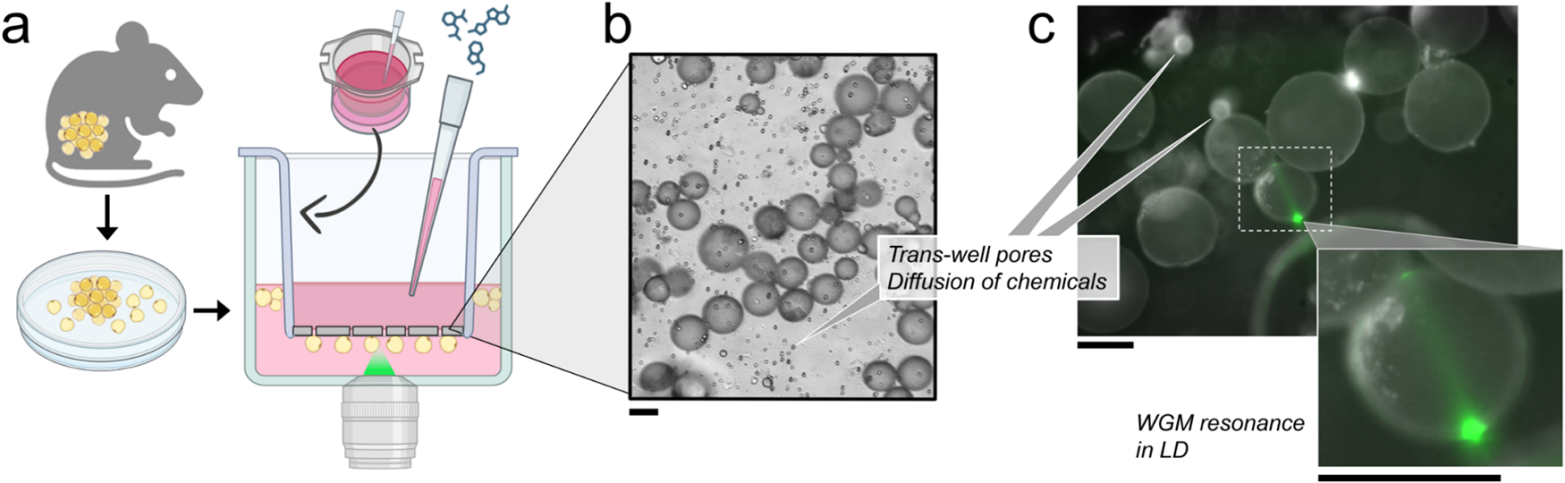
Transwell-based experimental setup with the typical size, surface coverage, and localized optical resonance in mature adipocytes isolated from mice. (a) Schematics of a workflow and a translucent transwell insert placed in a 12-well plate where the cells are homogeneously distributed under the surface. The plate was mounted on a stage-top incubator fitted on an inverted microscope. (b) A typical example of a bright-field (BF) image, showing cell distribution and size with visible 8 μm pores that enable diffusion and exchange of chemicals between the upper and lower chambers. (c) An example of a fluorescence image of adipocytes with the locally focused laser beam (in green) exciting WGM resonances in a vertical plane visible as a line and a smaller bright spot on the other side of the LD. Scale bar is 50 μm.

#### Optical Setup

Adipocyte LDs, as natural intracellular optical microresonators, were excited at their circumference using nanosecond pulsed (Opotek, OpoletteTM 355) and compact diode CW (Thorlabs) lasers, both set to a wavelength of λ = 532 nm, optimized for BODIPY laser dye excitation. To achieve the optimal lasing on live adipocytes below the threshold for photodamage, the energy per pulse of the pulsed laser with a 20 Hz repetition rate was typically 0.1–0.5 μJ (with an irradiance of *I*_pulse_ < 10^9^ W/cm^2^), while the power of the CW laser was 0.1 μW with an irradiance of *I*_pulse_ ≈ 10 W/cm^2^. The lasers were aligned in the back port of the microscope. For optimal focusing and detection of WGM resonances, a 20× objective (*NA* = 0.45) and exposure times of 0.5 to 5 s were used, respectively. WGM spectra were captured using an imaging spectrometer (Shamrock SR-500i, Andor) with a diffraction grating of 1200 lines/mm, resulting in a resolution of 0.07 nm. A 50 μm wide slit aperture was used at the spectrometer entrance for optimal concality and signal-to-noise ratio (S/N). Wide-field fluorescence imaging of cell organelles, LDs, plasma membrane, and nuclei was acquired simultaneously in separate fluorescence channels with a digital camera (sCMOS Zyla 4.2, Andor) using LED source illumination (CoolLED, pE-300 white), dichroics, and band-pass filters (all Semrock).

#### Image/Spectral Analysis

The measured spectra of pump-induced lasing or high S/N are the superposition of (differently polarized) transverse electric (TE) and/or transverse magnetic (TM) spectral eigenmodes/WGMs.^3^ Their positions were calculated according to the first-order radial modes approximation description,^50,51^ where each peak position was fitted with the following function

1 $$L\left(\lambda \right)=\sum_{i_{\mathrm{TM}},i_{\mathrm{TE}}}\frac{1}{2\pi }\frac{\Gamma }{{\left(\lambda -\lambda_{i_{\mathrm{TM}},i_{\mathrm{TE}}}\right)}^{2}+{\left(1/2\Gamma \right)}^{2}}$$

λ_*i*_TM_,*i*_TE__ are the spectral peak positions defined by the mode numbers (*m*), polarization, refractive index ratio, and the LD size, and Γ is a parameter specifying function width. We assumed that both internal and external refractive indices are constant and directly calculated the LD size. The assumption of constant refractive indices is justified in the continuation. The examples of spectral fits to the background-subtracted and normalized raw data in a time experiment are shown in Figures S1 and S2a. In cases of low S/N, where the spectra resembled a sinusoidal shape, we developed an empirical model that nicely fits the experimental data after the background signal. Such broadened spectra are typical for a large number of measured adipocytes with noncomplete sphericity and smoothness of the LD surface. The corresponding empirical model is

2 $$f\left(\lambda \right)=A\sin\left[(k\left(1-B{\left(\lambda -\lambda_{\min}\right)}^{2}\right)\lambda +\phi \right]$$

where *A* is the intensity of the WGM peaks, *k* is the wavenumber of the quasi-sinusoidal function at the left boundary of the measured spectral interval at λ_min_, and ϕ is the phase shift required to properly align the fitting function to the measured WGM resonances. *B* is the scaling constant to properly fit the nonlinear (quadratic) dependence of the spacing between consecutive resonant modes, the so-called free spectral range (FRS), with wavelength λ*. B* can be used to extract the refractive index dispersion with the wavelength Δ*n*(λ). Due to typically smaller Δ*n*(λ) than the measured peak position uncertainty in low S/N spectra obtained in multiple cases, we simplified the model. Since the WGM resonances are periodic in inverse wavelength, the model can be adapted as follows

3 $$f\left(\lambda \right)=A\sin\left[k^{\prime }\lambda^{-1}\right]$$

where *k*’ is a scaling constant that encodes the proportionality with FSR = 1/π*n*_eff_ *d*, where *n*_eff_ is the effective refractive index of LD potentially influenced by the surrounding medium through the evanescent field and *d* is the LD diameter. From the peak positions of the fitted model (example in Figure S2b), we calculated FSR between individual peaks across the spectra (λ_*m*+*i*_) to determine *d* and, consequently, the change in LD diameter (Δ*d*) over time using the equation

4 $$\frac{\sum_{i=1...N}\left(\frac{1}{\lambda_{m+i}}-\frac{1}{\lambda_{m+i-1}}\right)}{N}=\frac{1}{\pi n_{\mathrm{eff}}d}$$

The accuracy of calculated Δ*d* was validated optically (Figure S3), showing excellent correlation with our model under the assumption of a constant effective refractive index (*n*_eff_) throughout the experiment (Figure 2). This confirmed that Δ*d* can be precisely quantified well below the optical resolution limit using WGM fitting alone, whereas direct optical measurements are sensitive only to Δ*d* above ∼1 μm and are accompanied by large errors.

**Figure 2:**
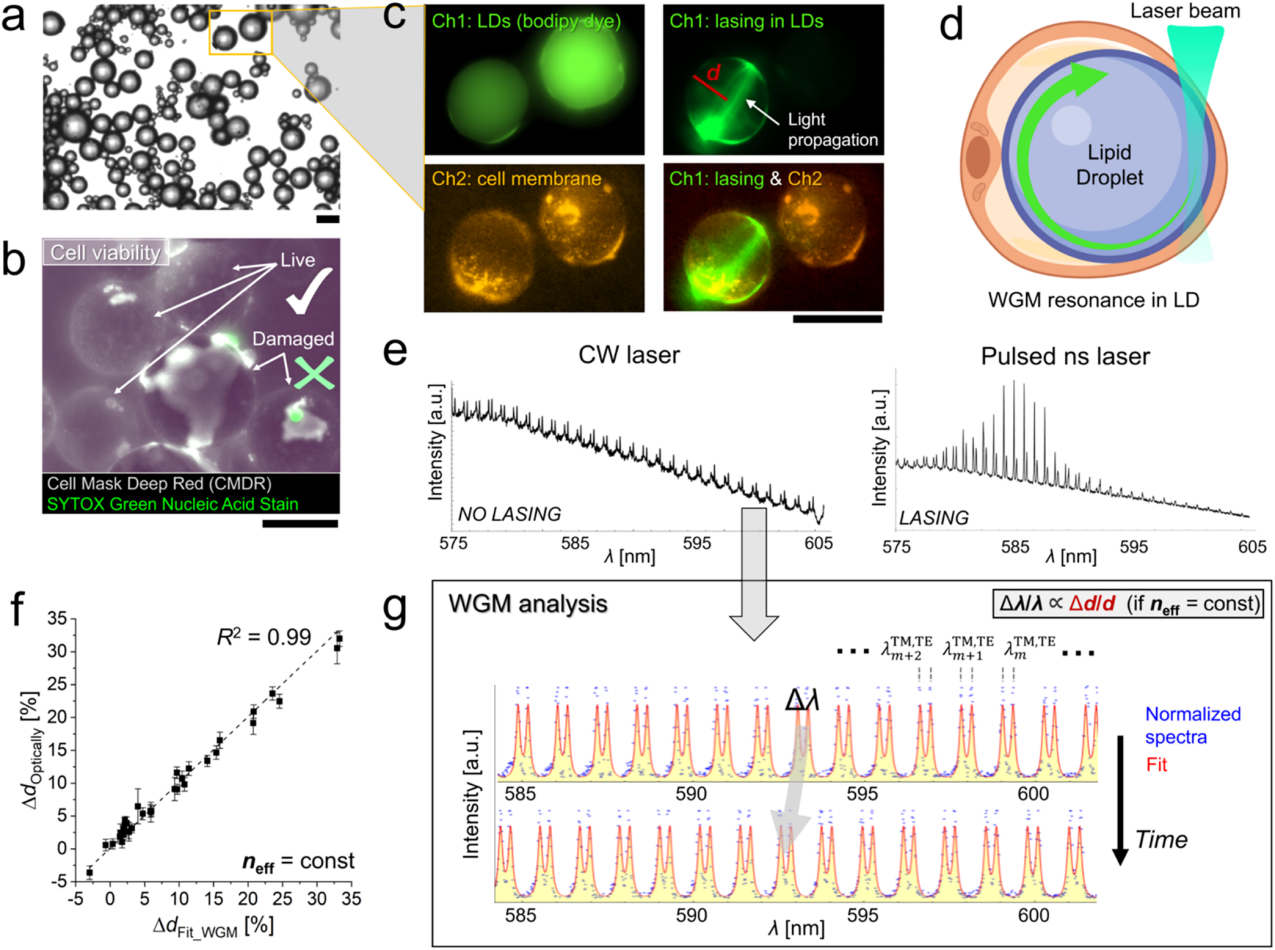
Workflow of ultraprecise adipocyte dynamics study using WGM sensing and analysis. (a) Bright-field (BF) image of isolated adipocytes. (b) Example of cell viability measurement with double staining used to evaluate both structural and functional integrity. Typically, damaged cells with the labeled nuclei (in green) are accompanied by ruptured cell membranes (more images in Figure S5b and Supporting Information Video S2). (c) Upper row: fluorescence of LDs and CW laser-induced light propagation at the LD circumference observed in the same channel (Ch1, in green). Bottom row: fluorescence of cell plasma membrane observed in other channels (Ch2, in orange) and the overlay of Ch1 (lasing) and Ch2. (d) Schematics of the laser excitation of the dye within the LD and the light circulation at the LD circumference forming WGM resonances. (e) Typical WGM spectra formed inside adipocyte LDs induced by CW laser (on the left) and pulsed ns laser (on the right). (f) Validation of the WGM fitting using optical measurements of the LD Δ*d* taken after 1 day showing a perfect correlation (*R* ^2^ = 0.99), where the *n*_eff_ was kept constant over time. (g) The following analysis of WGM resonance spectra (points in blue) with typical transverse magnetic (TM) and transverse electric (TE) eigenmodes, to quantify the position of spectral peaks (λ_m_) and hence the LD size (*d*) by using proper fitting (in red). The scale bar is 50 μm.

## Results and Discussion

### Experimental Approach for Nanometer-Precision Optical Sensing of Mature Adipocytes

Adipocytes isolated from mouse or human adipose tissue represent one of the most challenging biological systems for advanced microscopy techniques. Their study requires careful optimization of both experimental conditions and optical sensing to capture fast, dynamic processes. The unique properties of adipocytes—particularly their highly spherical shape and low density—contribute to their buoyancy in the cellular medium, necessitating a customized imaging platform, as previously demonstrated.^52^ On one hand, their near-perfect sphericity demands three-dimensional fluorescence imaging for accurate characterization of cell morphology and, critically, cell viability. On the other hand, their buoyancy and surface confinement require an optimized experimental setup, achieved here using a translucent ceiling transwell system (schematic in Figure 1a). This workflow enabled controlled administration of chemical reagents to stimulate metabolic activity while maintaining the immobility of nonadherent, mature adipocytes—essential for dynamic single-cell studies. We began with large-field-of-view (FoV) bright-field (BF) imaging to assess the quality of adipocyte isolation, evaluate ceiling coverage under the transwell, and determine cell size distribution (Figures 1 and 2a). Evenly distributed pores, averaging 8 μm in diameter and visible in fluorescence imaging (see arrows), facilitated solvent diffusion from the top to the bottom chamber, achieving complete mixing and concentration equalization within 1 h (Figure S4). Finally, fluorescently labeled LDs were illuminated at their circumference with a laser to excite the WGMs (Figure 1c).

Prior to measuring the LD size via optical resonances, we assessed adipocyte viability using plasma membrane staining (CellMask Deep Red) and nucleic acid staining (SYTOX Deep Red), enabling both direct and indirect detection of compromised membranes (Figure 2b). Variability in sample preservation across biological replicates—likely due to differences in AT from individual mice, resulted in adipocyte viability ranging from approximately 50 to 80% (Figure S5). Fluorescence staining also allowed us to evaluate the functional integrity and structural changes of adipocytes. The results presented in Figure S6 suggest potential cellular mechanisms involving plasma membrane remodeling, discussed in greater detail in the Supporting Information, Note A. Given the complexity of the biological system under study, we focused exclusively on mature, nondifferentiated adipocytes that maintained structural and functional integrity of both the plasma membrane and LDs throughout the duration of the experiment.

### Biological Assessment and Evaluation of LD Lasing

By precisely positioning the pump laser at the perimeter of the LDs, we excited the gain medium composed of Bodipy-based Pyrromethene 597 (Figure 2c). The resulting fluorescent light was confined along the LD boundary via total internal reflection, as illustrated schematically in Figure 2d, producing distinct spectral features known as WGMs (Figure 2e and Supporting Video S1). Depending on the excitation source—pulsed or continuous-wave (CW) laser—different gain conditions were achieved, resulting in either true lasing or cavity-modified fluorescence.^5^ In both cases, the spectra exhibited sharp lines corresponding to WGM resonances; however, under lasing conditions, these lines were narrower and more pronounced (Figure 2e, right panel). Surpassing the lasing threshold required high concentrations of the lipophilic fluorescent dye and particularly high-energy nanosecond pulsed laser excitation, reaching up to μJ per pulse. These settings were found to induce local photodamage in adipocytes via photoablation, as detailed in ref 53. High-Q WGMs below the damage threshold were achievable only in adipocytes with structurally compromised LDs that exhibited near-perfect sphericity. This geometry, along with a smooth and polished LD surface, is known to support sharp resonance peaks^54^ and enable subnanometer resolution.^14^ In practice, however, the shape and surface smoothness of LDs are influenced by mechanical and tension forces exerted by the surrounding cytoskeleton—primarily a diffuse cortical actin network^55^ and intermediate/vimentin filaments.^56^ These forces can locally disrupt the LD sphericity and surface integrity, significantly reducing the achievable Q-factor.

Only a small number of adipocytes emitted measurable WGMs when excited by a nanosecond pulsed laser at a pulse energy of *E*_pulse_ ≈ 500 nJ and a peak power density of *I*_pulse_ ≈ 10^9^ W/cm^2^. These levels are approximately 10 times higher than those used in our previous study,^14^ approaching the photoablation damage threshold.^53^ Surprisingly, a cost-effective, low-power CW diode laser outperformed the pulsed laser in both spectral sensitivity and safety, as demonstrated on the same adipocyte (Figure S7). We identified the CW diode laser as the most optimal light source suitable for 100% safe WGM-based biosensing in mature adipocytes. Its operating parameters—carefully set just below the threshold for photochemical or photothermal effects (Figure S7)—consistently produced detectable WGMs (spectra in blue). The SNR varied depending on the concentration of the gain medium and local deviations in sphericity and surface smoothness at the LD boundary of individual adipocytes (Figures 2e, left panel; S2).

We acquired and analyzed WGM resonances induced by a CW laser. Spectra with higher SNR, exhibiting clearly distinguishable TM and TE spectral eigenmodes (Figures 2e, left panel, 2g and S2a), were fitted using the first-order radial mode approximation.^50^ In contrast, spectra with lower SNR and significant spectral broadening (Figure S2b) were fitted using an empirical model, as detailed in the Materials and Methods section. Due to the lower Q-factor of the detected eigenmodes in both cases—compared to the high-Q WGM lasing spectra typically observed in embedded microspherical resonators—we could not achieve the same level of precision in the peak uncertainty ( $\sigma_{\lambda_{i_{\mathrm{TM}},i_{\mathrm{TE}}}}$ ) and hence in the microresonator size measurement previously reported.^14^ By applying spectral fitting to eigenmodes with profiles resembling Gaussian shapes, and using a well-defined model for peak position error (dependent on spectrometer resolution, sampling density, and SNR),^57^ we calculated the $\sigma_{\lambda_{i_{\mathrm{TM}},i_{\mathrm{TE}}}}$ ,^58^ and thereby the resolution in peak position. For the spectra shown in Figures 2g and S2a—with an SNR of approximately 40 and spectral width *w ≈* 0.15 nm—the WGM peak uncertainty was calculated to be $\sigma_{\lambda_{i_{\mathrm{TM}},i_{\mathrm{TE}}}}$ ≈ 0.005 nm, while for the spectra with lower SNR of approximately 10 shown in Figure S2b, it was calculated to be $\sigma_{\lambda_{i_{\mathrm{TM}},i_{\mathrm{TE}}}}$ ≈ 0.027 nm. The detailed calculation of the uncertainties is presented in Supporting Note B.

The positions of WGM resonances are sensitive to changes in both size (*d*) and effective refractive index (*n*_eff_), which is the refractive index of the LD (*n*_LD_) slightly modified by the refractive index of the nearby cell cytoplasm (*n*_cell_). *n*_cell_ can vary over time and has a typical spread across a population of cells of up to Δ*n*_cell_ ≈ 0.0006.^59^ Because the variation of *n*_cell_ within a single cell is typically smaller than that observed across a population of physically and metabolically heterogeneous cells, and because WGM resonances shifts are considerably less sensitive to changes in *n*_cell_ than in *n*_LD_, the potential Δ*n*_cell_ could not be detected given the spectral peak uncertainty and the corresponding LD size resolution of 3.5 nm described at the end of this section. If Δ*n*_cell_ is overestimated to be 0.001, the measured LD size change would be Δ*d* ≈ 2.2 nm for a typical 60 μm LD. Furthermore, WGMs in live cells cannot sense changes in extracellular environment due to cytoplasmic thickness of the order of a micron. WGMs are sensitive to changes only within ∼100 nm of the lipid droplet surface, corresponding to the typical penetration depth of the resonator’s evanescent field, and the sensitivity at this distance is already markedly reduced compared with the surface. Similar arguments apply to *n*_LD_, where molecular exchange, and hence, changes in the chemical composition in LDs are far too small to produce detectable variation in *n*_LD_. This conclusion is further supported by optical validation of Δ*d* performed on a small fraction of adipocytes with optically measurable Δ*d* (only after prolonged exposure to the lipolytic agent) (Figure 2f). The results showed excellent correlation between both methods, with *n*_LD_ (*n*_eff_) kept constant over time in the WGM fitting and analysis. The validation not only confirms Δ*d* as the primary cause of the observed WGM spectral shifts, but also demonstrates the ability to calculate Δ*d* with precision well below the optical resolution limit using WGM fitting alone.

In addition to Δ*d*, spectral shifts may also arise from local deviations of the lipid droplet (LD) from perfect sphericity, potentially coupled to cellular rotation between consecutive time points. To assess such deviations, we analyzed the LD size at various points along its circumference by altering the laser position (Figure S8). The observed dispersion in LD size, Δ*d* ≈ 10 nm, measured across multiple cells, was smaller than the typical spectral changes recorded during time-lapse measurements following lipolytic agent administration (Figure 3). Therefore, morphological heterogeneity is expected to have a minimal impact on the accuracy of quantifying biological heterogeneity and LD dynamics over hour-scale observations. Nonetheless, it is essential to carefully account for these factors.

**Figure 3:**
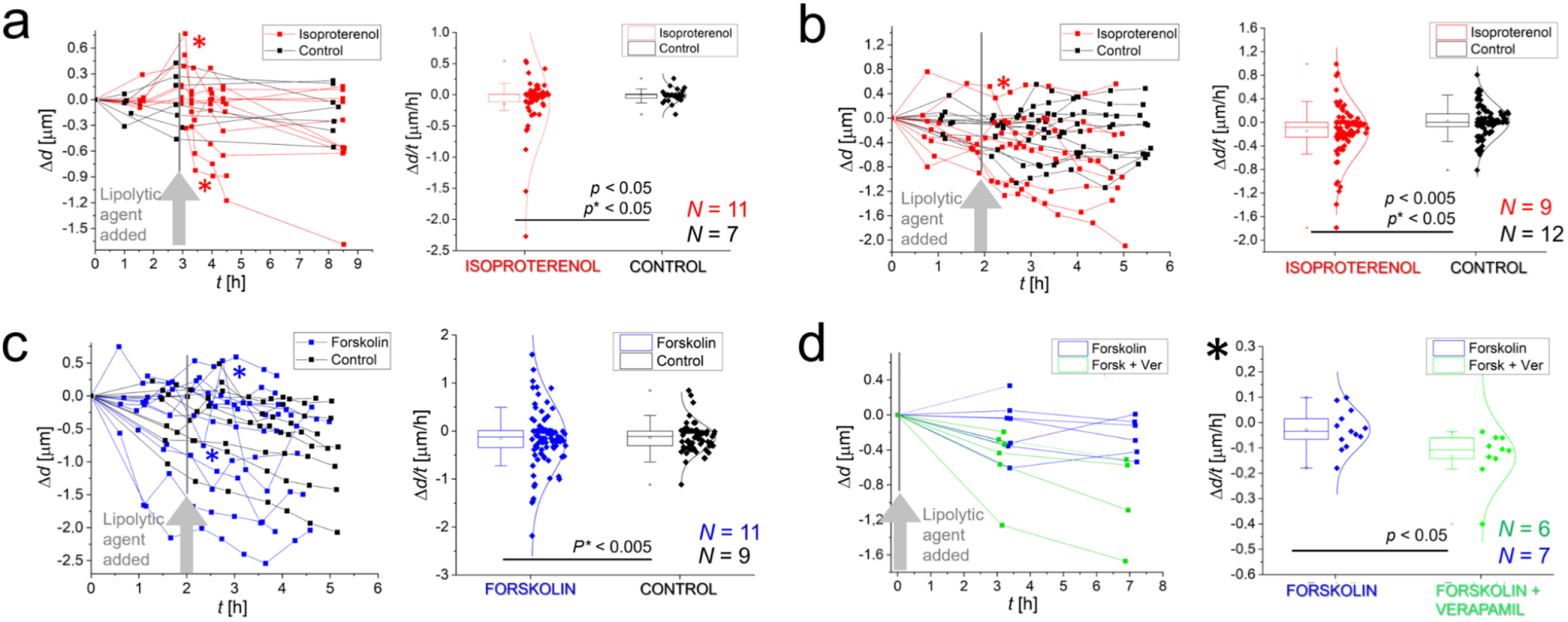
Measurements of LD size dynamics to external stimuli introduced by isoproterenol and forskolin. (a) Isoproterenol-induced LD size change (Δ*d*) and LD rate of size change (Δ*d*/*t*) between consecutive time points performed on multiple cells over a longer time with a longer sampling interval. The results show significantly different population variances and suggestively different population means of the Δ*d*/*t* between the exposed cells and the control (*p* = 0.05—two-sample *t* test for null hypothesis that the means are not the same; p* < 0.05—Levene’s test for population variance). (b) Second biological replicate of isoproterenol-induced LD Δ*d* and Δ*d*/*t* performe*d* on multiple cells with a shorter sampling interval over time. The results show significantly different population variances and means of the Δ*d*/*t* between the exposed cells and the control (p* < 0.05; *p* < 0.05). (c) Results of forskolin-induced LD Δ*d* and Δ*d*/*t* performed on multiple cells in the time experiment. The results show significantly different population variance of the Δ*d*/*t* between the exposed cells an*d t*he control (p* < 0.005). (d) Results of verapamil-boosted LD Δ*d* and Δ*d*/*t* performed on multiple cells in the time experiment with a long sampling interval. The results show significantly different population means of the Δ*d*/*t* between the cells exposed with one and two lipolytic agents (*p* < 0.05).

Mechanical drift, minor cell movement, and environmental fluctuations were evaluated as potential sources of error and were found to have no significant impact on accuracy. Positional shifts during long-term experiments remained well below the imaging field of view and were routinely corrected. Because each individual measurement lasted only a few seconds under stable incubator conditions, these factors were negligible within this time frame and did not affect the recorded data.

To estimate the achievable repeatability in the measured *d*, it is necessary to account for potential scatter and noise in the multiple spectra acquired from a stable LD during a typical time-lapse experiment. WGM spectra recorded over a 3 h interval with 0.5 h time steps on a stable LD revealed minimal peak scatter, approximately *w*_s_ = 0.04 nm (Figure S9). Since the observed scatter *w*_s_ slightly exceeds the WGM peak uncertainties from spectral fitting, $\sigma_{\lambda_{i_{\mathrm{TM}},i_{\mathrm{TE}}}}$ , we used *w*_s_ to estimate the achievable uncertainty of the measured Δ*d*. Applying the relation (Δ*d*)_min_ = *dw*_s_/λ, we obtained an uncertainty—and thus a resolution, of approximately 3.5 nm, which is 2 orders of magnitude better than what is achievable with confocal microscopy. In our experimental setup, the maximum applicable numerical aperture (*NA* = 0.8) provided an optical resolution of approximately 0.5 μm. However, such measurements are accompanied by a large relative error (Figure S3), particularly for submicron- to micron-scale changes in Δ*d*, which have been shown to dominate LD dynamics (Figure 3). Consequently, the optical sensing of lipid droplet size dynamics with high precision is practically impossible.

### Adipocyte Heterogeneity and Rapid Metabolic Response Revealed by the Rate of LD Size Change

Having demonstrated the ability to accurately quantify the LD size, we now apply this capability to investigate the temporal dynamics of adipocytes. Specifically, we monitored the LD size over time and evaluated its response to lipolytic stimulation with agents such as forskolin and isoproterenol.

The initial time points reveal heterogeneous behavior among individual mature adipocytes, even prior to stimulation (Figure 3). Some cells exhibit lipogenesis (an increase in LD size), others undergo lipolysis (a decrease in the LD size), while the remainder maintain a steady-state (homeostatic) condition. The variability in our results is consistent with recent studies employing single-cell transcriptomic profiling.^32,60^ These studies demonstrate high heterogeneity in adipocyte tissues, revealing diverse cell subtypes associated with distinct physiological states. Some subtypes exhibit high lipogenic capacity, whereas others are linked to lipolysis.^60^

Capturing multiple baseline time points before administering the lipolytic agent was essential for establishing a reference, enabling the reliable quantification of LD kinetics following stimulation. The sparse and variable response to external stimuli reflects diverse and uneven activation across the cell population. Analysis of LD size dynamics/change (Δ*d*) in stimulated adipocytes (Figure 3, left graphs) shows a slightly broader distribution skewed toward lipolysis. Given the inherent heterogeneity observed even in control samples, additional measurements are needed to assess the statistical significance of differences in LD size and, by extension, metabolic activity between exposed and nonexposed cells. Nevertheless, in nearly all biological replicates (each comprising approximately 10 adipocytes)—we observed individual cases within the stimulated group showing substantial and rapid Δ*d* across consecutive time points (Figure 3, asterisks; Figure S10, arrows). An increased rate of LD size change (Δ*d/t*) was observed within a ∼30 min interval (Figure 3, right graphs).

Stimulation with isoproterenol at different sampling intervals (Figure 3a,b) resulted in a statistically significant increase in both the population mean and the variance of the Δ*d*/*t*. In contrast, stimulation with forskolin (Figure 3c) did not yield a significant difference in the population mean but did reveal a significant increase in the variance of Δ*d*/*t*. In treated cells, Δ*d*/*t* reached up to approximately 1 μm/h, compared to ∼0.5 μm/h in control cells. From this, we can estimate the molar flux of molecules involved in lipolysis and lipogenesis. For a 60 μm-sized adipocyte, the average molar flux—based on observed size changes, was calculated to be approximately 4 × 10^–7^ mol/m^2^/s. This corresponds to the transport of ∼10^9^ molecules/s across the LD surface, assuming an average triglyceride (TG) molecular volume of 2 nm^3^.^61^ To contextualize these results, the estimated molar flux aligns to that reported in a recent study using a conventional lipolytic calorimetric assay on primary adipocytes.^62^ In that study, a lipolytic rate of 160 nmol/well/h corresponds to a molar flux of 1.3 × 10^–7^ mol/m^2^/s, assuming that the total LD surface area in confluent cells is approximated by the surface area of the assay well. Our findings provide a quantitative assessment of the rate and extent of transient metabolic responses to lipolytic agents, revealing a complex feedback mechanism that regulates both lipolysis and LD integrity.

By comparing the lipolytic activity of isolated mature adipocytes from our study with that of model adipocytes differentiated from 3T3-L1 cells^35,39,63^ under identical external stimuli, we observed a slightly weaker response in LD dynamics. Several hours of exposure to isoproterenol-induced size changes of up to 2 μm (Figure 3a,b), corresponding to a functional readout of metabolic/lipolytic efficiency ranging from an average of 0.002 to 0.007 μm^3^/min per μm^2^ surface area, assuming a typical LD diameter of 60 μm. In contrast, the lipolytic efficiency in model adipocytes exposed to isoproterenol was up to 10-fold higher (average 0.02 μm^3^/min/μm^2^).^63^ Exposure to forskolin induced less pronounced size changes compared to the control (Figure 3c), up to ∼ 0.5 μm, translating into a lipolytic efficiency of ∼0.001 μm^3^/min/μm^2^. This was several-fold lower than in model adipocytes exposed to forskolin (0.004 μm^3^/min/μm^2^, assuming a typical LD diameter of 5 μm).^35,39^ The higher lipolytic efficiency in model adipocytes can be attributed to their greater responsiveness to lipolytic stimuli compared with isolated mature adipocytes, which may be metabolically compromised and possess a lower surface-to-volume ratio that limits signal transduction.^63^ Nevertheless, high Δ*d*/*t* values in individual adipocytes measured within a 30 min window indicate short-period bursts of lipolytic flux after stimulation with both agents (Figure 3, asterisks), comparable to those observed in model adipocytes. These results are consistent with the current understanding of LD dynamics in model adipocytes and further reveal the complexity and heterogeneity of primary adipocytes, which are more directly translatable to in vivo biology and disease contexts.

In Figure 3d, we present another example of sensing stimulated adipocyte metabolism using a combination of drugs. The sapling interval was rather long, resulting in a different metric for Δ*d*/*t* (see the asterisk), which is not comparable to the rest. However, forskolin and verapamil (in green) appear to have a greater effect on lipolysis and its rate than forskolin alone (in blue). Verapamil may enhance forskolin-induced lipolysis indirectly by blocking calcium (Ca^2+^) influx channels.^64^ Reduced intracellular Ca^2+^ levels help sustain cAMP activity, which is essential for lipolysis. Conversely, elevated Ca^2+^ concentrations are known to suppress cAMP signaling^65^ and consequently inhibit lipolysis in adipocytes.^66^ Further biological replicates are needed to confirm the role of verapamil in the iodoprosyl alcohol in this process.

Our new approach, employing nonphototoxic CW laser, provides a valuable enhancement to existing lipolytic assays by offering a faster, more precise, and cost-effective method for sensing adipocyte responses to lipolytic agents. A key advantage is its ability to quantify responses at the single-cell level, revealing biological heterogeneity and potential intercellular interactions that conventional techniques may overlook.

### WGM Resonances Enable Fast Diagnostics of Cell Viability

Throughout the study, fluorescence imaging revealed alterations in the adipocyte plasma membrane morphology and integrity at the single-cell level (Figures 2b and S5). Interestingly, we noticed that these changes correlate with changes in the WGM resonances. A more detailed analysis (Figure 4) demonstrated that spectral changes occurred significantly earlier than those detectable by visual inspection or the SYTOX Deep Red viability assay. While the CW laser was optimal for measuring metabolic activity in live adipocytes, we employed a pulsed laser in this context because of its superior spectral sensitivity in distinguishing live from damaged or dead cells. This includes the sensitivity to the change in both spectral intensity and shape, which are less pronounced with a CW laser (Figure S11). To minimize the risk of photoablation damage from a pulsed laser during measurements, we used lower doses and irradiances per pulse (*E*_pulse_ < 500 nJ; *I*_pulse_ < 10^9^ W/cm^2^). These conditions were generally insufficient to generate WGM resonances in live adipocytes, as shown in Figure S7.

**Figure 4:**
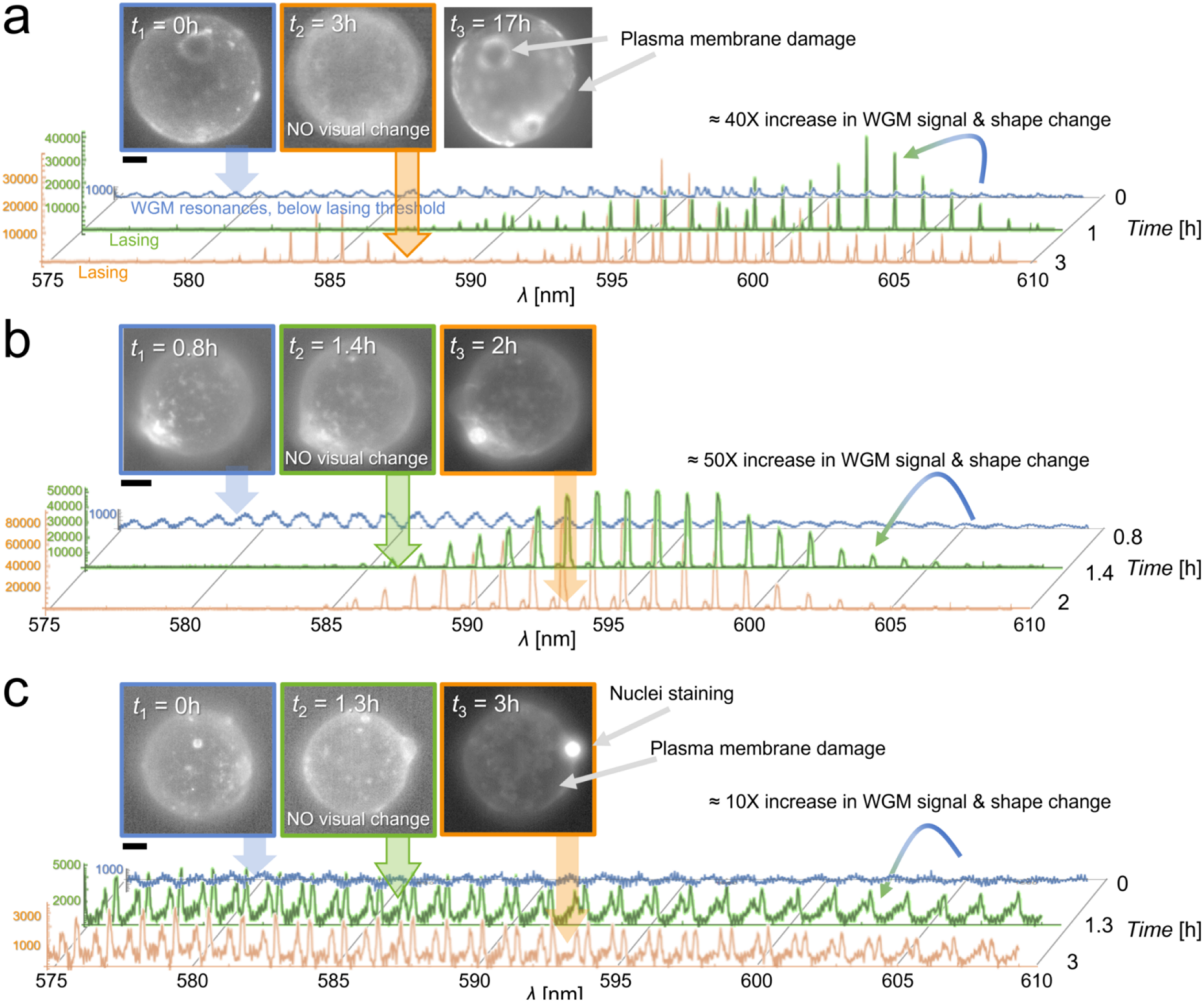
WGM spectra-based rapid diagnostics of adipocyte viability. (a) Fluorescence images of the same cell at three time points, stained with plasma membrane CellMask Deep Red dye and the corresponding changing WGM spectra color-coded in blue, green, and orange. (b, c) Two more examples on adipocytes which undergo plasma membrane rupture observed additionally with the SYTOX Deep Red nuclei staining dye as a viability assay. Again, spectral change observed between first and second time points was detected before any morphological change (image outlined in green) and before being diagnosed with the viability assay (image outlined in orange). The time-lapse of the adipocyte damage, including the excretion of LD content, is provided in Supporting Video S2. The scale bar is 10 μm.

As highlighted, WGM spectral analysis detected cellular damage prior to visual confirmation via fluorescence imaging—either directly through plasma membrane integrity (Figure 4a) or indirectly via nuclear staining (Figure 4b). Characteristic spectral transitions were observed as WGM resonances shifted from a nonlasing (subthreshold) to a lasing mode (Figure 4a,b, blue to green spectra, indicated by the colored arrows). The full time-lapse sequence of adipocyte membrane rupture and partial LD release into the extracellular space for the example presented in Figure 4b is available in Supporting Video S2.

WGM measurements are highly sensitive to ultrasmall changes in lipid droplet (LD) morphology, their physical properties, and the characteristics of the surrounding microenvironment. These measurements likely detect cytoskeletal remodeling—particularly actin and microtubule disruption, leading to LD relaxation toward a more spherical and smoother surface morphology, a process that precedes plasma membrane damage and subsequent cell apoptosis. This underscores the importance of mechanical signals, alongside biological markers, in assessing cell viability, as recently demonstrated.^67^ A detailed analysis of the spectra shown in Figure 4c and presented in Figure S12 revealed a slight drift between the second and third time points, accompanied by a measurable increase in transverse electric (TE) and transverse magnetic (TM) mode splitting. This shift suggests a reduction in the refractive index of the LD’s immediate surroundings (*n*_cell_), primarily composed of cytoplasm. Spectral fitting indicated a decrease in Δ*n*_cell_ of approximately 0.028 ± 0.002, from *n*_cell_ = 1.367 to *n*_cell_ = 1.339, assuming *n*_LD_ = 1.47.^68^ These findings align with both spectral and visual evidence of compromised plasma membrane integrity, where cytoplasmic dilution due to extracellular fluid influx lowers *n*_cell_. The increased mode splitting is likely attributable to the TM mode’s heightened sensitivity to perturbations in the near-field environment, owing to its deeper penetration into the evanescent field at the resonator surface, as described in prior experimental and theoretical studies.^69,70^ Another example of this phenomenon is shown in Figure S13, where mode splitting correlates strongly with the plasma membrane rupture. In this case, the refractive index of the cytoplasm decreased by Δ*n* = 0.032 ± 0.002, from approximately *n* = 1.392 to 1.36, indicating significant hydration and cytoplasmic loss. Through precise WGM spectral quantification in adipocytes, this study not only captures rapid metabolic dynamics but also reveals the physiological state of cells prior to conventional viability assays.

Time-lapse experiments across multiple biological replicates also revealed that damaged cells—initially identified by spectral changes and later confirmed by plasma membrane disruption (Figure 4), exhibited a loss of LD dynamics, maintaining a constant LD size over time (Figure 5). The distinction between damaged and viable adipocytes is clearly demonstrated by comparing the distributions of LD size dynamics (black vs orange). These findings reinforce that LD dynamics, monitored over several hours, serve as a reliable indicator of cellular state and viability.

**Figure 5:**
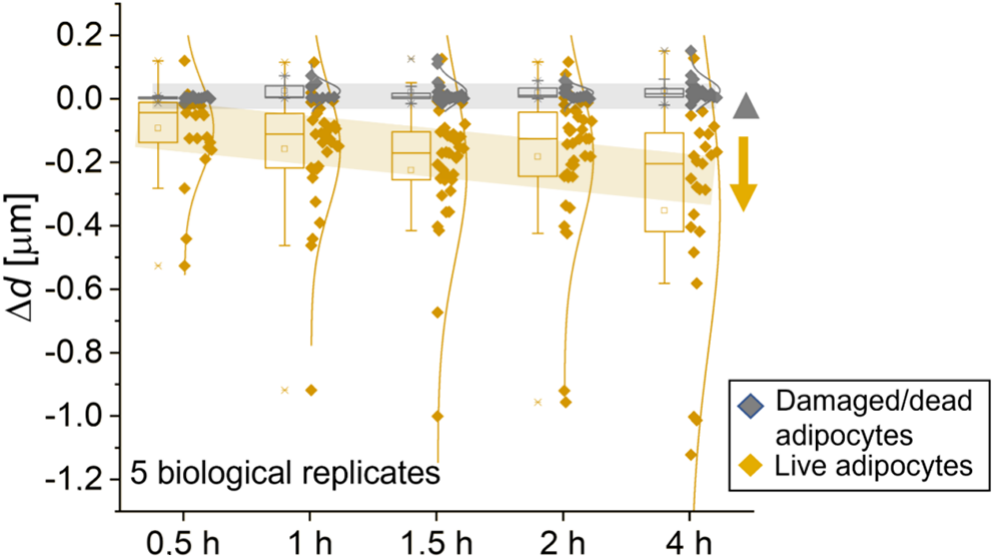
A comparison and a clear difference in the dynamics of LDs size change (Δ*d*) between live and damaged adipocytes obtained from 5 biological replicates, each analyzing several cells. The sample size for each biological replicate was between 3 and 8.

## Conclusions and Future Outlook

We present a novel experimental methodology for precisely sensing adipocyte size dynamics with exceptionally high-spatial (subnanometer) and temporal (subminute) resolution. This approach builds on our earlier study, where we demonstrated lasing inside live cells for the first time.^14^ We systematically investigated the physical and biological aspects of photonic sensing in live, mature adipocytes, highlighting both its capabilities and its limitations. Due to the nonspherical and uneven morphology of LDs in live adipocytes, conventional methods for generating WGM resonances proved inadequate. Instead of using a pulsed nanosecond laser—which requires high lasing powers and risks photoinduced damage, we implemented a cost-effective, low-power CW laser. Although this setup did not yield the maximum Q-factor or spectral resolution typical of pulsed lasers, it was not a limiting factor. The temporal spectral noise was comparable to the peak position uncertainty from spectral fitting, resulting in a precision of approximately 3.5 nm—2 orders of magnitude better than the optical resolution achievable with confocal microscopy. Using precise WGM spectral characterization and appropriate fitting models, we quantified biological and morphological heterogeneity in adipocytes and captured rapid metabolic responses to external lipolytic stimuli. These responses were measurable through changes in the LD size and molecular flux rates. Moreover, WGM spectral shifts proved to be a promising tool for rapid diagnostics of the cellular state and viability. Our results establish a proof-of-concept that significantly advances current lipolytic and viability assays by enabling faster, more cost-effective, and single-cell–level assessment of primary adipocyte dynamics and heterogeneity, which are indispensable for understanding genuine metabolic responses. Unlike conventional assays, which rely on bulk cell populations and cannot resolve interactions between individual cells, our methodology enables such an analysis. If adapted for high-throughput imaging and further combined with emerging single-cell RNA-sequencing techniques,^32,60,71^ this complementary approach holds broad potential for investigating the mechanisms of metabolic and obesity-related diseases at both cellular and tissue scales. It would substantially increase sample size and statistical power, improving precision in characterizing biological and morphological heterogeneity and enabling more robust insights into fast transient and early kinetic–dynamic processes within adipose tissue.

## Supplementary Material







## Data Availability

The raw data underlying this study are openly available in the Zenodo repository at DOI: 10.5281/zenodo.17806820.

## Notes

Figure S1: WGM spectra preparation and analysis to calculate the change in LD size; Figure S2: examples of spectral fits to differently shaped WGM spectra; Figure S3: validation of LD size change with optical measurements; Figure S4: diffusive transport of lipolytic agents through a transwell system used in our study; Figure S5: examples of adipocyte preservation and viability after isolation from mice visceral fat; Figure S6: evaluation of the viability of adipocytes; Figure S7: performance of pulsed and CW pump laser sources for safe WGM-based biosensing applications on adipocytes; Figure S8: measurement of the LD sphericity; Figure S9: measurement of the spectral repeatability on a stable LD; Figure S10: transient effect on individual LD in the rate of size change (Δ*d*/*t)* after isoproterenol stimulation; Figure S11: comparison of WGM spectra-based rapid diagnostics of adipocyte viability using pulsed and CW laser sources; Figure S12: analysis and fitting of WGM spectra in a perturbed adipocyte with a significant spectral shape change; Figure S13: an example of significantly increased TE and TM mode splitting in the WGM spectra due to plasma membrane rupture; Supporting Note A: comment on plasma membrane remodeling; and Supporting Note B: detailed description of WGM peak uncertainty calculation (PDF)
Typical WGM spectra formed inside adipocyte LDs (MP4)
Time-lapse of the adipocyte damage, including the excretion of LD content (MP4)
