# Supplementary material for: Nanometer-Precision Tracking of Adipocyte Dynamics via Single Lipid Droplet Whispering-Gallery Optical Resonances
Source: ACS Sens. 2025 Dec 29;11(1):511–21. doi: 10.1021/acssensors.5c03272 (PMC12836352; doi:10.1021/acssensors.5c03272)
Supplement: Supplementary file 1 [file se5c03272_si_001.pdf]

Nanometer-precision tracking of adipocyte dynamics via single lipid droplet whispering-gallery optical resonances

Rok Podlipec[1*], Ana Krišelj[1], Maja Zorc[1], Petra Matjan Štefin[2,3], Siegfried Usaar[4,5], Matjaž Humar[1,6,7]

1 Department of Condensed Matter Physics, Jozef Stefan Institute, Jamova 39, SI-1000 Ljubljana, Slovenia

2 Department of Biochemistry and Molecular and Structural Biology, Jozef Stefan Institute, Jamova cesta 39, SI-1000 Ljubljana, Slovenia

3 Jozef Stefan International Postgraduate School, Jamova cesta 39, SI-1000 Ljubljana, Slovenia

4 Research Unit Adipocytes & Metabolism (ADM), Helmholtz Diabetes Center, Helmholtz Zentrum München, Germany; Research Center for Environmental Health GmbH, Neuherberg 85764, Germany

5 German Center for Diabetes Research (DZD), 85764, Neuherberg, Germany

6 Faculty of Mathematics and Physics, University of Ljubljana, Jadranska 19, SI-1000 Ljubljana, Slovenia

7 CENN Nanocenter, Jamova 39, SI-1000 Ljubljana, Slovenia

Corresponding author: rok.podlipec@ijs.si

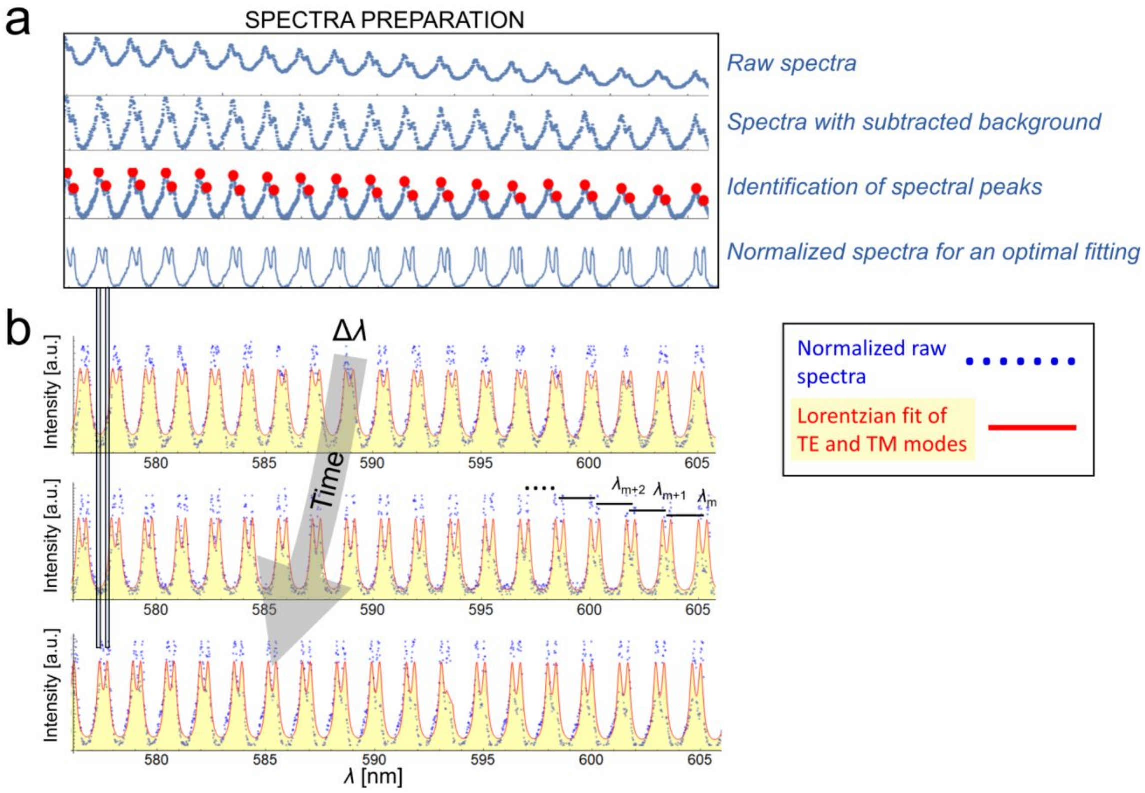

Figure S1. Consecutive steps of WGM spectra preparation and analysis to calculate change in LD size. (a) Spectra preparation for the optimized fitting. (b) Fitting of normalized spectra by using first order radial mode approximation description and Lorentzian function for TE and TM eigenmodes (equation 1) in the time experiment.

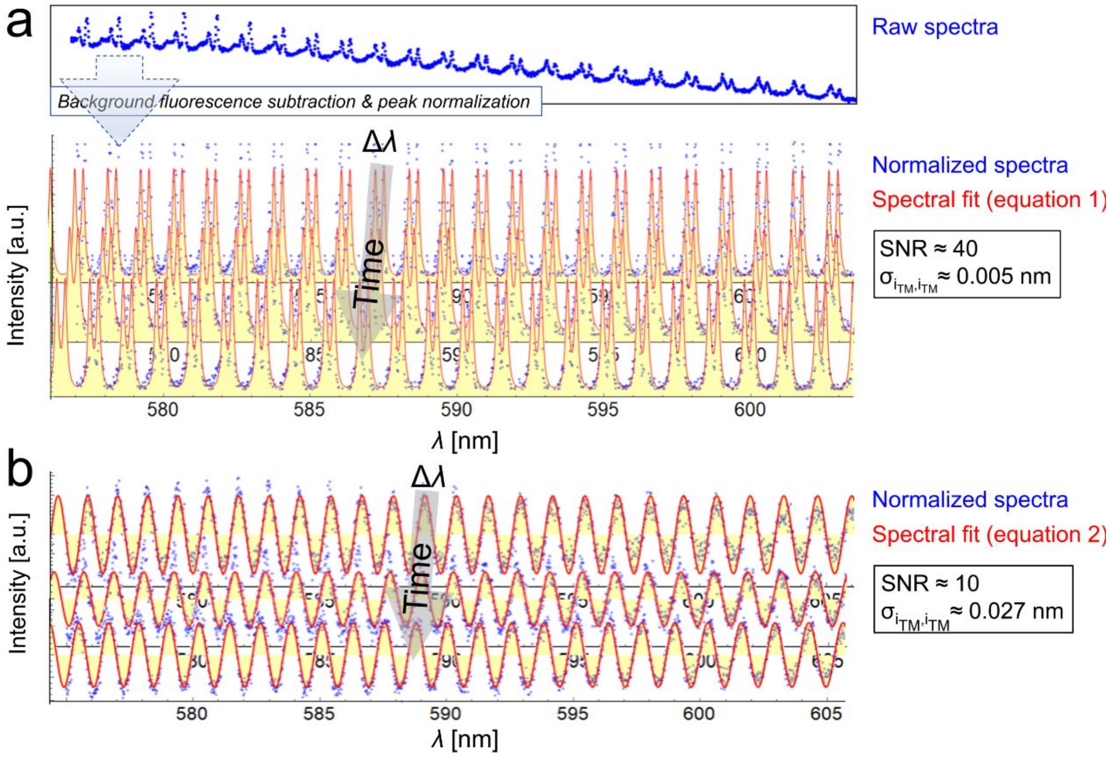

Figure S2. An example of different spectral fits to differently-shaped, background-subtracted and normalized raw WGM spectral data in a time experiment. (a) The workflow for precise spectral fitting and analysis of distinctly observed TM and TE eigenmodes providing the spectral peak uncertainty of $\sigma_{iTM,iTM} \approx 0.005$ nm for ultraprecise characterization of adipocyte size. (b) Precise spectral fitting and analysis of the WGM spectra with an extensive spectral broadening providing the spectral peak uncertainty of $\sigma_{iTM,iTM} \approx 0.027$ nm.

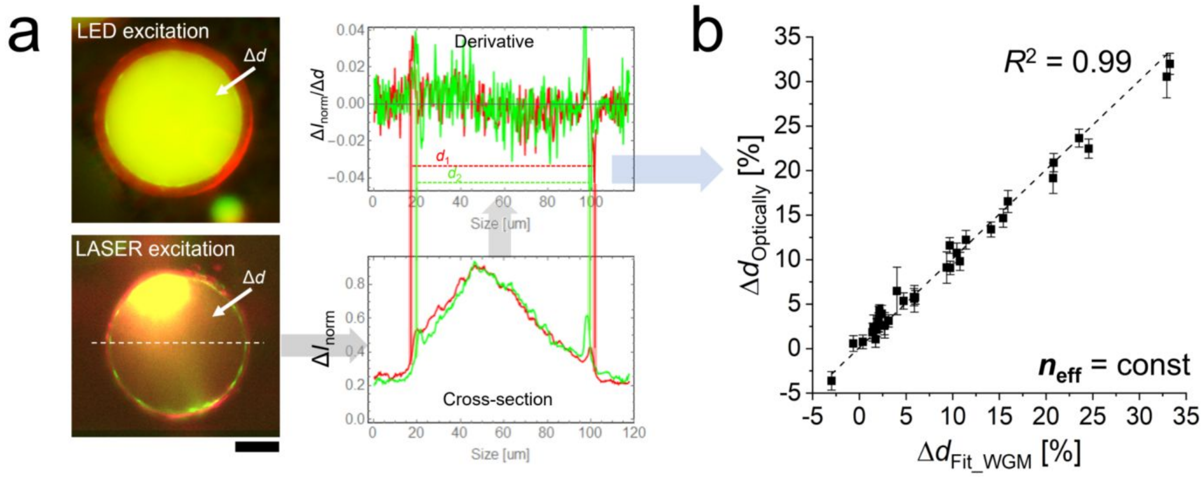

Figure S3. Optical measurements of LD size change (Δd) feasible in adipocytes with prolonged lipolytic activity. (a) An example of a significant decrease in LD size after one day, measured through the fluorescence signal of Pyromethene 597, artificially color-coded in red and green (left), and by image analysis of the size change obtained from the derivative of the intensity cross-section profile. (b) Comparison of optical measurements of Δd (in %) with values calculated from WGM fitting, under the assumption of a constant n_{eff} over time, revealed excellent correlation across the entire measured range (R^2 = 0.99).

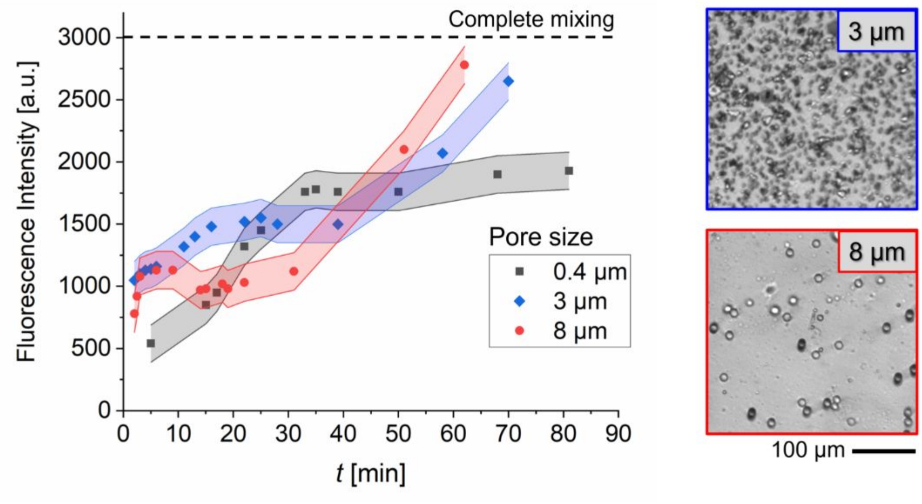

Figure S4. Diffusive transport of small molecules through different-sized pores in a trans-well system to reach complete mixing between both chambers. Faster transport of molecules through smaller 3 μm pores as compared to 8 μm pores in the first 30 min is attributed to the much higher density of smaller pores, as seen in the right images. The Trans-well with the smallest pores (0.4 μm in gray) did not achieve complete mixing within 90 minutes and was thus not used in our study. On the other side, both 3 μm and 8 μm trans-well chambers were appropriate for our multi-hour dynamics study on adipocytes. The color-coded bands show the error of the measurements.

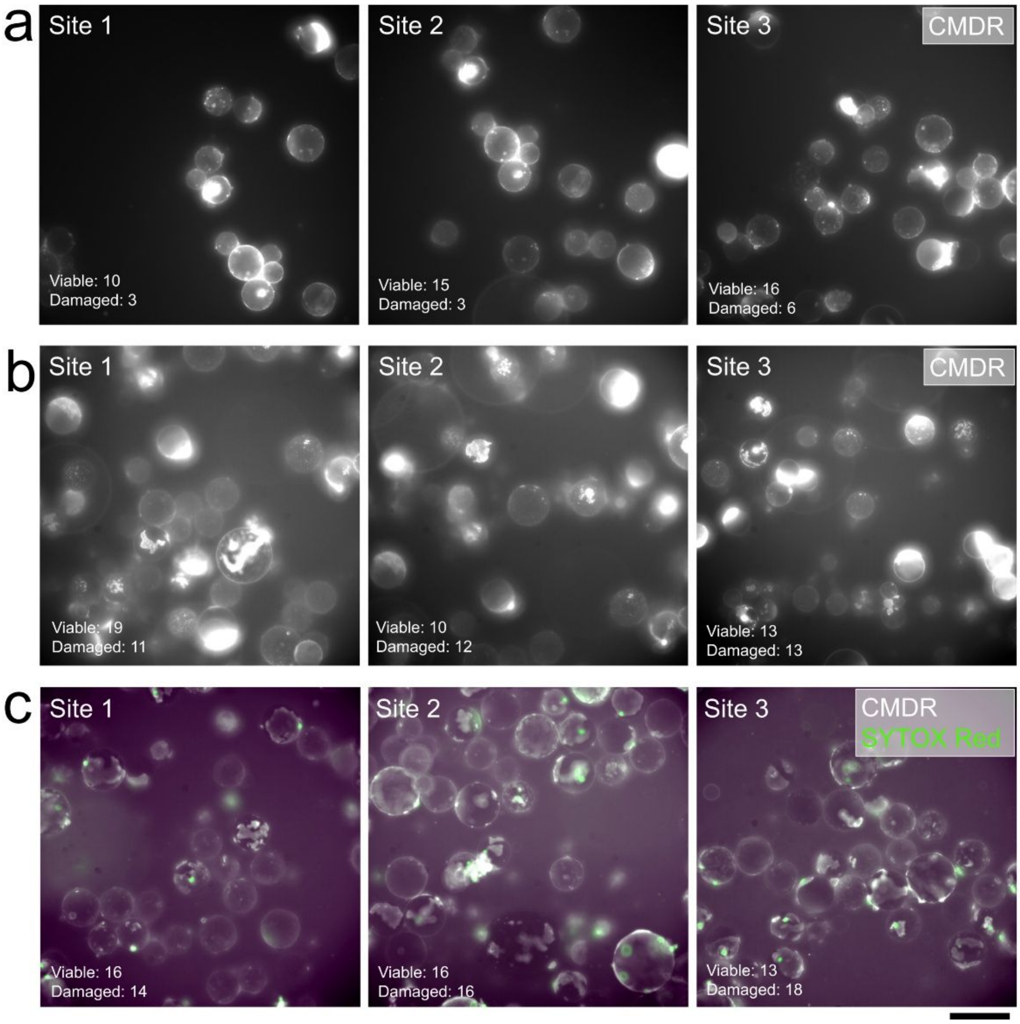

Figure S5. Adipocyte preservation and viability measurements after being isolated from visceral AT presented on different biological replicates (a-c). a) An estimated viability of 70-80% using CellMask Deep Red plasma membrane stain to track membrane rupture and localized accumulation of the stain. b) An estimated viability of 50-70% using the same stain. c) An estimated viability of 40-60% using the combination of CellMask Deep Red (in gray) and SYTOX Red nucleic acid stain (in green), with the distinct features nicely colocalized on the damaged structures. Scale bar is 100 μm.

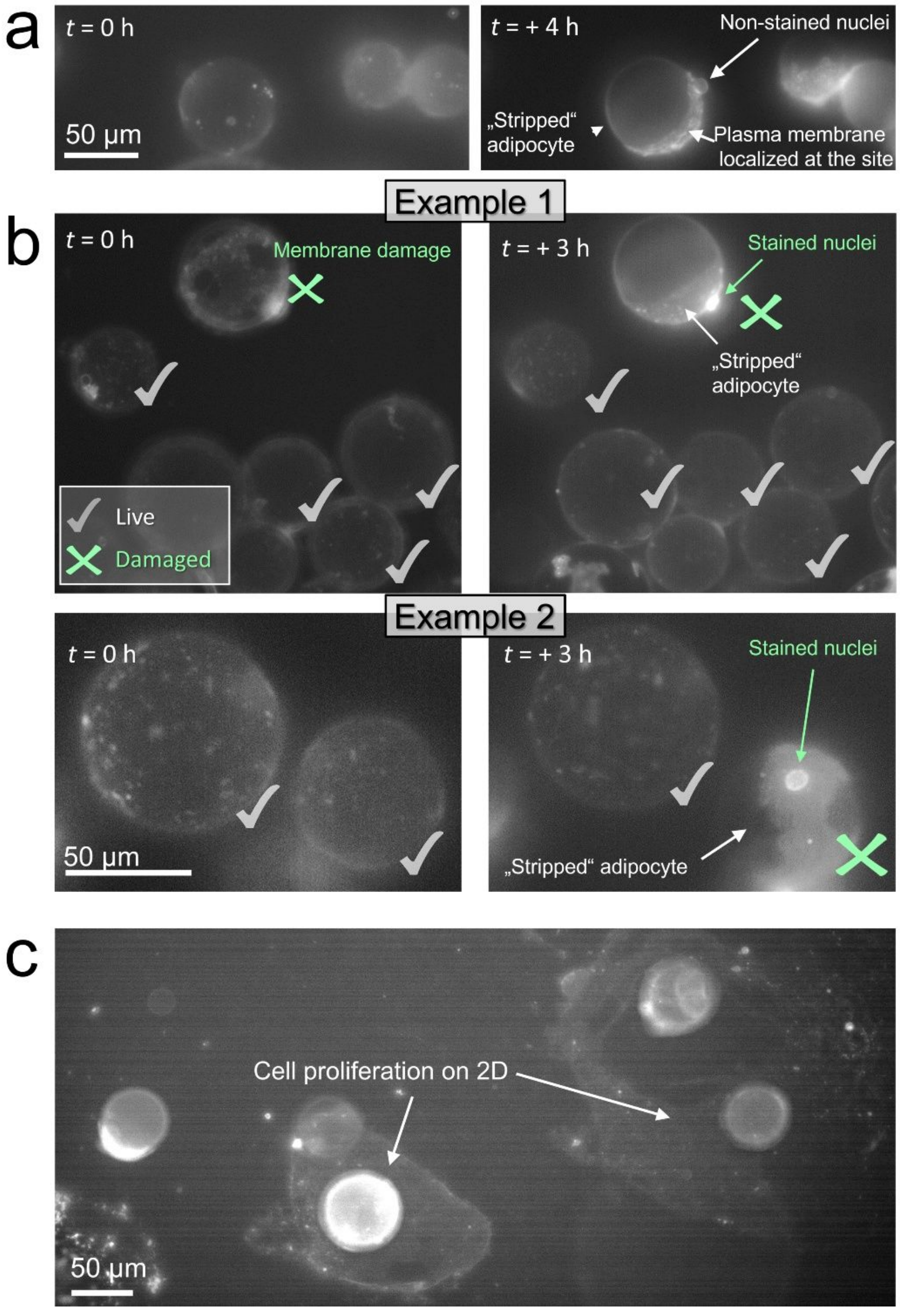

Figure S6. Evaluation of the viability of isolated mature adipocytes for further LD dynamics experiments. (a) Ruptured plasma membrane with possibly retained structural integrity due to non-stained nuclei. (b) The two exemplary cases where the remodeled/ruptured plasma membrane correlates with nuclear labeling, indicating cellular damage/death (marked in green). Adipocytes with a homogenously stained plasma membranes (checkmarks) remain

highly viable, as indicated by the live/dead assay. (c) Cell proliferation on the transwell surface indicates adipocyte dedifferentiation. Imaging was performed using a 20× objective (*NA* = 0.45) with fluorescent labeling of the plasma membrane (CellMask Deep Red, Invitrogen) and nuclei (SYTOX Deep Red).

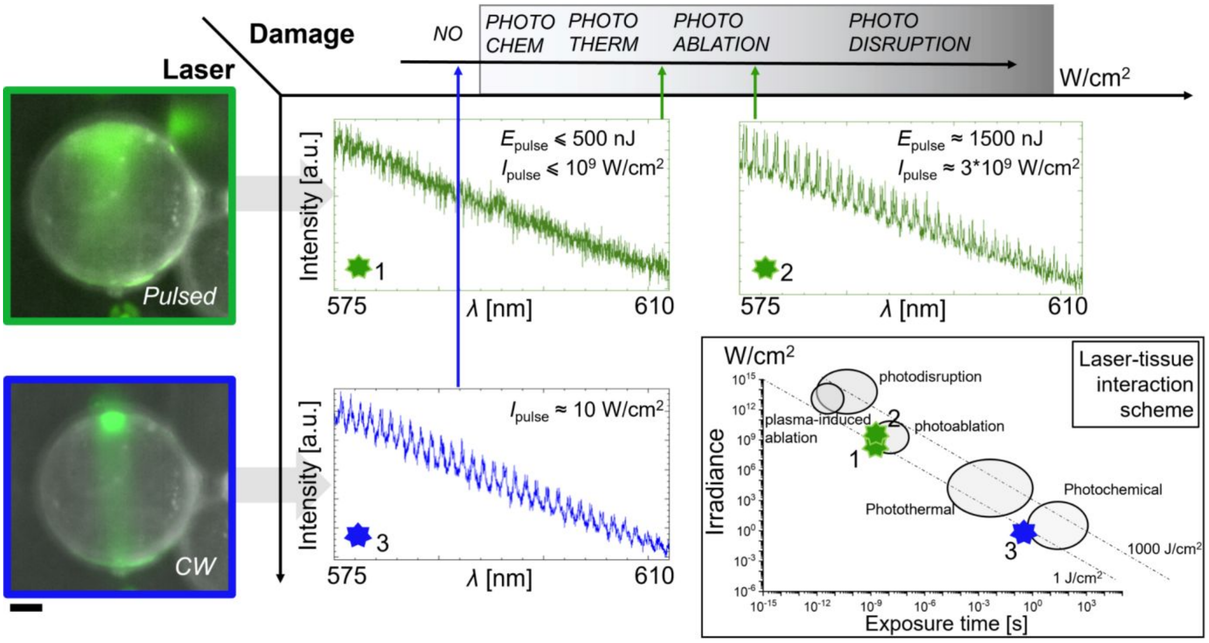

Figure S7. Performance of pulsed (ns) and CW pump laser sources for safe WGM-based biosensing applications on mature adipocytes. Due to the non-ideal sphericity and smoothness of LDs in live adipocytes, which are locally disturbed by the cytoskeleton and the imbalance of surrounding mechanical forces, high laser doses/irradiance per pulse (E_{pulse}/I_{pulse}), capable of inducing a photoablation-damaging effect (green arrow), are commonly required to introduce WGM lasing on adipocytes (2 - green spectra on the right). By introducing an alternative approach using a cost-efficient CW laser, slightly lower performance in spectral resolution and SNR was achieved (3 - blue spectra), but with negligible damaging effects, as shown with the blue arrow and schematically depicted in the laser-tissue interaction map on the bottom right (adapted from [1]). Scale bar is 10 μm.

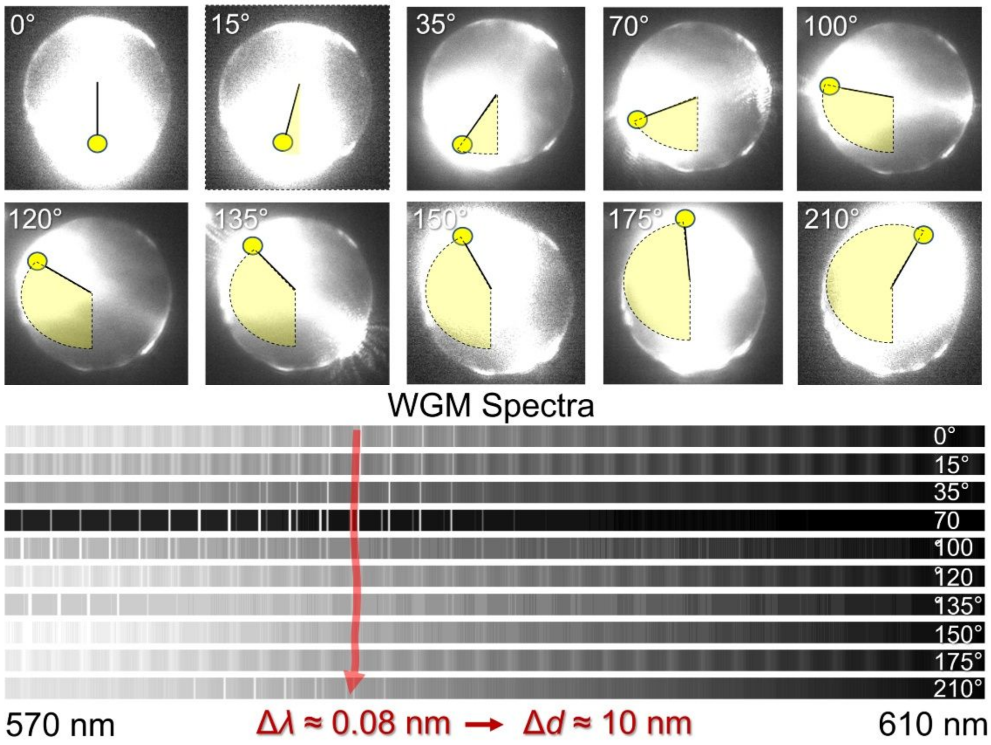

Figure S8. Measurement of the LD sphericity by changing the pump laser position around its circumference. Acquired spectra at different positions, from angle 0° to 210°, reveal deviation from complete sphericity for up to $\Delta\lambda$ = 0.08 nm (red arrow), which accounts for the diameter change of up to Δd = 10 nm, lower than typical changes induced by external stimuli.

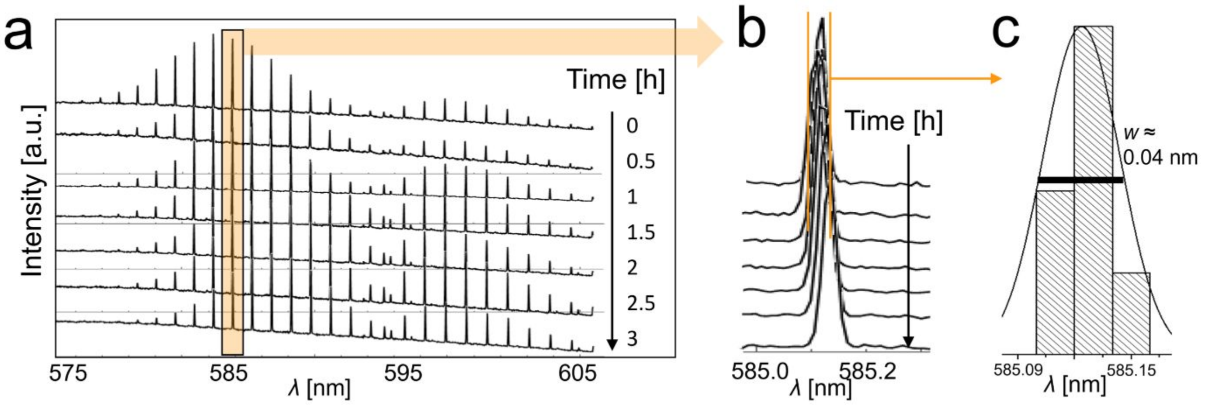

Figure S9. Measurement of the scatter of the multiple spectra acquired from a stable LD throughout a typical time experiment using a pulsed laser. We used a pulsed laser because it provides WGMs with a higher Q-factor than is achievable with a CW laser, thereby enabling

greater measurement precision. (a) WGM spectra performed in a 3-hour time interval with 0.5-hour time steps. b) A slight scatter and a directional trend (red shift) of the spectra as shown on a single eigenmode at λ = 585.13 nm. c) Distribution of peak positions gathered from multiple spectra.

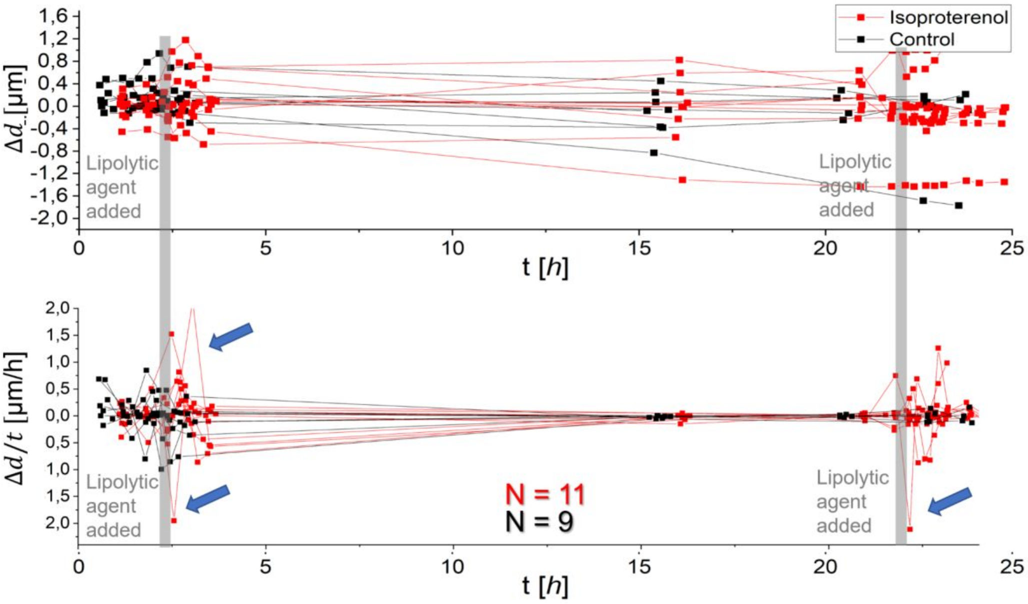

Figure S10. Adipocyte metabolic response via measurements of LD size (Δd) and rate of size change ($\Delta d/t$) to external stimuli induced by isoproterenol (connected data points in red). The data reveal adipocyte heterogeneity and a rapid, transient effect on individual adipocytes (marked with the blue arrow), where the rate of Δd oscillates significantly more than in control.

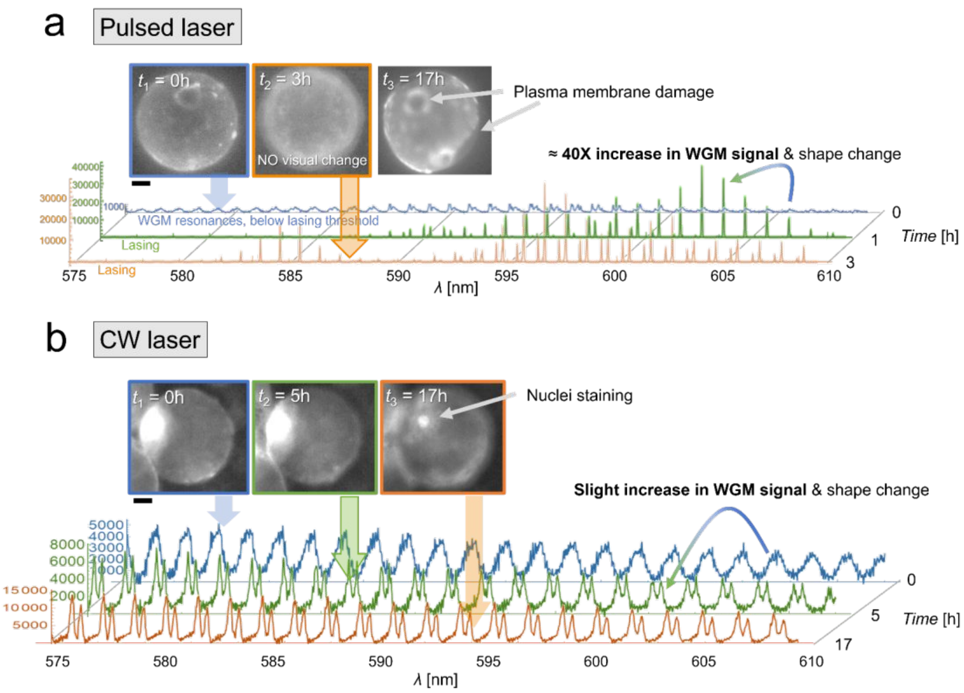

Figure S11. Comparison of WGM spectra-based rapid diagnostics of adipocyte viability using pulsed and CW laser sources, demonstrating superior spectral sensitivity with the pulsed laser. (a) Fluorescence images of the same cell at three time points, stained with the plasma membrane dye CellMask Deep Red, together with the corresponding WGM spectra induced by pulsed laser excitation, color-coded in blue, green, and orange. (b) Fluorescence images of the same cell at three time points, stained with CellMask Deep Red in combination with the nuclear viability dye SYTOX Deep Red, together with the corresponding WGM spectra induced by CW laser excitation, color-coded in blue, green, and orange. Scale bar: 10 µm.

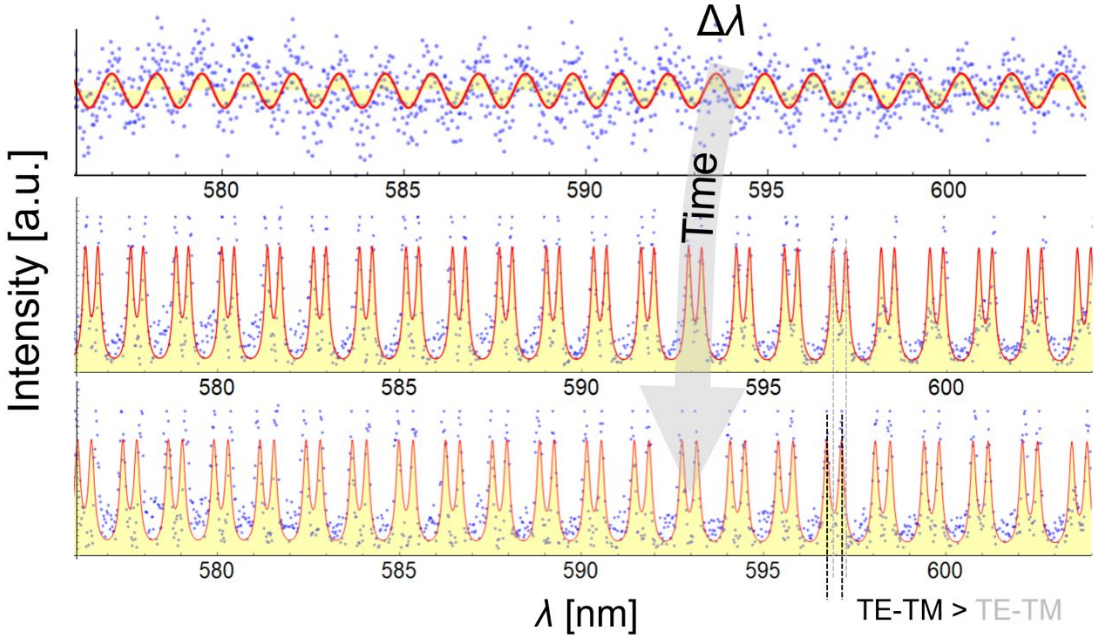

Figure S12. Detailed analysis and fitting of WGM spectra (in red) with an empirical (upper panel; equations 3 and 4) and exact (lower panels; equation 1) model for the measurement of LD size in a perturbed adipocyte. A significant spectral shape change was observed between the first and second time points, indicating cellular damage or plasma membrane rupture, which was later confirmed optically (see Figure 5c). A detailed analysis has also revealed a slight but measurable increase in TE and TM mode splitting (black and gray dashed lines), characterized by a decrease in the refractive index in the cellular cytoplasm, indicating dilution induced by plasma membrane rupture.

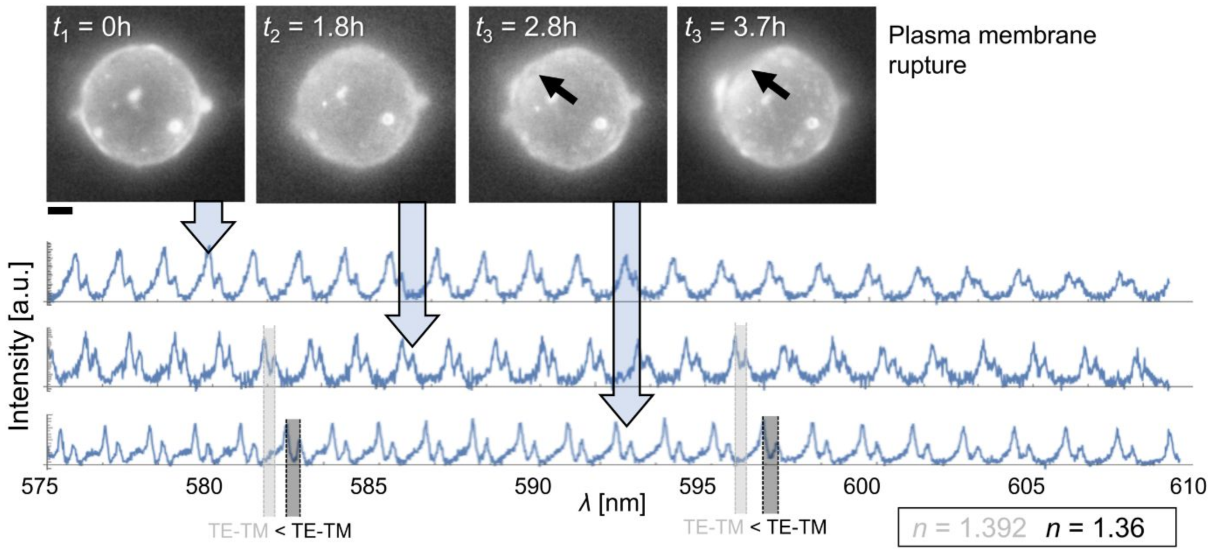

Figure S13. An example of significantly increased TE and TM mode splitting in the WGM spectra is shown between the second and third time points (see the width of the gray bars showing the splitting). This spectral change, characterized by a measurable decrease in the refractive index of the cellular cytoplasm around the LD, is correlated with plasma membrane rupture, as nicely observed in the last time point (see the black arrow). Scale bar is 10 µm.

Supplementary Note A

In certain cases of cellular disruption, where the plasma membrane appeared localized to one side of the "stripped" adipocyte, the membrane seemed to retain structural integrity. This was evident from the presence of unstained nuclei (Figure S5a), which under normal conditions would be stained (Figure S5b). Such observations suggest a potential, albeit unconventional, mechanism for preserving the function of core cellular components under mechanical, physical, or metabolic stress. In contrast, lipid droplets (LDs), once partially secreted, exhibited no dynamic activity, indicating arrested metabolism and/or cellular damage, as discussed later. Plasma membrane remodeling may therefore reflect not only cellular damage but also contribute to adipocyte dedifferentiation [2], as indicated by cell proliferation in 2D culture (Figure

S5c). Given the complexity of the biological system under study, we focused exclusively on mature, non-differentiated adipocytes that maintained both plasma membrane and LD integrity. In this context, staining of both nuclei and plasma membrane was essential.

Supplementary Note B

By applying spectral fitting to eigenmodes with profiles resembling Gaussian shapes, and using a well-defined model for peak position error (dependent on spectrometer resolution, sampling density, and SNR) [3] we calculated the uncertainty ($\sigma_{\lambda_{i_{TM},i_{TE}}}$) [4], and thereby the resolution in peak position. This uncertainty is expressed by the following equation:

$$\sigma_{\lambda_{i_{TM},i_{TE}}} = \frac{\sqrt{\Delta\lambda_s W}}{\text{SNR}}\sqrt{\frac{\Delta}{F(\Lambda)}},$$

where $\boldsymbol{\Delta\lambda_s}$ is the wavelength sampling step ($\boldsymbol{\Delta\lambda_s}$ = 0.02 nm); W is related to the spectral full width at half maximum (FWHM; w) as $W \approx w/2.35$; SNR is defined as the maximum signal value at each eigenmode divided by the spectral noise; Δ is a numerical constant representing the confidence limit for a one-parameter fit (Δ = 1 corresponding to a 68% confidence interval or "1σ" error); $F(\Lambda)$ is a dimensionless factor derived from the integral of $(\partial \boldsymbol{S}_{\text{eigenmode}}/\partial \boldsymbol{\lambda}_{i_{TM},i_{TE}})^2$ over the λ-acquisition range (Λ), where $\boldsymbol{S}_{\text{eigenmode}}$ denotes the spectral profile of the individual eigenmode. Given that our acquisition range spans the full spectrum, $\boldsymbol{F}^{1/2}$ assumes a value of approximately 0.8 under the assumption of Gaussian noise [3]. Prior to calculating $\boldsymbol{\sigma}_{\lambda_{i_{TM},i_{TE}}}$, the equation was slightly adjusted to account for oversampling in $\boldsymbol{\Delta\lambda_s}$ relative to the spectrometer resolution (0.06 - 0.07 nm). This oversampling effectively reduced the number of independent data points across the acquisition range, leading to a modest increase in peak position uncertainty, as supported by analytical and numerical evaluations in study [3]. For the spectra shown in Figure 2g and Figure S2a—with an SNR of approximately 40 and $w \approx 0.15$ nm—the WGM peak uncertainty was calculated to be $\boldsymbol{\sigma}_{\lambda_{i_{TM},i_{TE}}} \approx 0.005$ nm.

References

(1) Keiser, G. Light-Tissue Interactions. In *Biophotonics: Concepts to Applications*; Keiser, G., Ed.; Springer Nature: Singapore, **2022**; pp 169–221. DOI:10.1007/978-981-19-3482-7_6

(2) Kim, J.; Park, K.-Y.; Choi, S.; Ko, U. H.; Lim, D.-S.; Suh, J. M.; Shin, J. H. Ceiling Culture Chip Reveals Dynamic Lipid Droplet Transport during Adipocyte Dedifferentiation via Actin Remodeling. *Lab Chip* **2022**, *22* (20), 3920–3932. DOI:10.1039/D2LC00428C

(3) Bobroff, N. Position Measurement with a Resolution and Noise-limited Instrument. *Review of Scientific Instruments* **1986**, *57* (6), 1152–1157. DOI:10.1063/1.1138619

(4) Urbančič, I.; Arsov, Z.; Ljubetič, A.; Biglino, D.; Strancar, J. Bleaching-Corrected Fluorescence Microspectroscopy with Nanometer Peak Position Resolution. *Opt Express* **2013**, *21* (21), 25291–25306. DOI:10.1364/OE.21.025291
